# Most and Least Preferred Colours Differ According to Object Context: New Insights from an Unrestricted Colour Range

**DOI:** 10.1371/journal.pone.0152194

**Published:** 2016-03-29

**Authors:** Domicele Jonauskaite, Christine Mohr, Jean-Philippe Antonietti, Peter M. Spiers, Betty Althaus, Selin Anil, Nele Dael

**Affiliations:** 1 Institute of Psychology, University of Lausanne, Lausanne, Switzerland; 2 Global R&D, AkzoNobel, Slough, United Kingdom; 3 IRP Chair in Spinal Cord Repair, Laboratory Courtine, Swiss Federal Institute of Technology (EPFL), Lausanne, Switzerland; University of Melbourne, AUSTRALIA

## Abstract

Humans like some colours and dislike others, but which particular colours and why remains to be understood. Empirical studies on colour preferences generally targeted most preferred colours, but rarely least preferred (disliked) colours. In addition, findings are often based on general colour preferences leaving open the question whether results generalise to specific objects. Here, 88 participants selected the colours they preferred most and least for three context conditions (general, interior walls, t-shirt) using a high-precision colour picker. Participants also indicated whether they associated their colour choice to a valenced object or concept. The chosen colours varied widely between individuals and contexts and so did the reasons for their choices. Consistent patterns also emerged, as most preferred colours in general were more chromatic, while for walls they were lighter and for t-shirts they were darker and less chromatic compared to least preferred colours. This meant that general colour preferences could not explain object specific colour preferences. Measures of the selection process further revealed that, compared to most preferred colours, least preferred colours were chosen more quickly and were less often linked to valenced objects or concepts. The high intra- and inter-individual variability in this and previous reports furthers our understanding that colour preferences are determined by subjective experiences and that most and least preferred colours are not processed equally.

## Introduction

Ask anyone about his or her preferred colour, and you are likely to receive an instant answer. Ask anyone about his or her preferred colour for particular objects, the answer might take slightly longer to occur. Having ever observed customers (or being a customer oneself) in a hardware store selecting any other colour but white for an interior wall, the efforts and selection procedure can become an ordeal. Professional colour consultants and trend watchers offer us their help in selecting the “right” and currently fashionable colours for particular objects and situations within seemingly boundless possibilities (e.g. [1–2]). Publicly available colour polls tell us what other people prefer (e.g. [3–4]). Considering that humans can distinguish about 10 million colours [5], it is not surprising that choosing the right colour can be difficult. So far, little is known about exactly which and why certain colours are liked and others disliked given that colour selection is such a pervasive everyday activity.

There are three basic perceptual attributes of colour: hue, colourfulness and brightness [6]. Hue is what laypersons refer to as colour (e.g. blue, red, orange etc.). For monochromatic light, hues correspond to different wavelengths within the visible part of the electromagnetic spectrum (e.g. from red, 700 nm, to violet, 400 nm). At the retinal level, three different types of cones are sensitive to different ranges of the visible spectrum (i.e. Long (L), Medium (M) and Short (S) cones are sensitive mainly to the reddish, greenish and bluish thirds of the spectrum, respectively). Within the visual cortex, colour is processed by cone-opponent mechanisms (S-(L+M): blue-yellow dimension, L-M: red-green dimension) [6]. Colourfulness (vividness) refers to an attribute of visual colour perception according to which a colour shows more or less of its hue. At the lower extreme of this dimension lie the achromatic colours grey, black, and white. Brightness is the light-dark dimension of the colour according to which a colour appears more or less light. Various models exist that describe colour using different definitions or calculations of these three attributes. Some are perceptually uniform, and resemble human colour perception (e.g. Munsell, CIE Lab), others are not perceptually uniform, and often depend on the hardware used to produce the colour (e.g. RGB, HSB, CMYK).

Colour preferences have long been explained through hue effects. In an early study, Eysenck [7] reported that participants preferred blue hues most and yellow hues least when asked to rank given colour patches in the order of preference. Similar results were reported from subsequent studies [8–11] despite using varying measurement techniques (e.g. rating pre-selected colours or asking participants to imagine colours). However, hue alone is insufficient in explaining colour preferences and other colour dimensions related to brightness and colourfulness are important as well, which was acknowledged already in 1926 [12]. Palmer and colleagues [13] recently reviewed colour preference studies and concluded that, in general, more colourful (vivid) colours are preferred to less colourful colours [14–16] while lighter colours are preferred to darker colours [14, 17]. Hence, all three colour attributes or dimensions are linked to colour preferences and need to be taken into account when exploring this subject.

A common procedure to assess colour preferences is through the presentation of pre-selected colour patches, either as physical coloured chips (e.g. [18]) or on a computer monitor (e.g. [15, 19–20]). This procedure indirectly implies that participants’ most as well as least preferred colours are part of the sample. Since the number of colours in the standard colour samples rarely exceeds 32 (e.g. [15]), the findings on colour preferences are only true for the presented colour samples and might differ for other samples. Alternatively, participants choose their preferred colours from a large array of possible colours within given hardware constraints [13]. In line with this suggestion, Fortmann-Roe [21] investigated colours that Twitter users chose in their social themes. When designing the appearance of their Twitter page, users use a colour picker to select any colour or colour combinations they want. After analysing over half a million Twitter accounts, the authors concluded that users chose shades of blue, cyan and red most often while almost never shades of green. People also chose relatively bright colours, but saturation (a colourfulness attribute) varied a lot between genders and individuals. It should be noted here that each colour dimension was analysed separately without controlling for potential influences from the other colour dimensions (but see [22]). Thus, in real life choice-settings, colour preferences seem to be more diverse than previous studies have suggested.

Another underdeveloped question is whether general colour preferences hold for various contexts (i.e. for specific objects, occasions, etc.). In an early study, Holmes and Buchanan [23] asked participants to imagine their favourite colour in general and also their preferred colours for different contexts (e.g. blouse, walls). The majority of participants identified various shades of blue (e.g. blue, navy blue) as their overall favourite colours, but identified other colours for different contexts. For example, shades of red were preferred for female blouses while white or very light colours for walls. Because colours were imagined, independent effects of hue, colourfulness or brightness could not be measured. Important here, this early work suggests that favourite colours in general may not be the same colours as favourite for specific objects. In a similar imagination study, participants liked blue colour the most overall, but white for different rooms (bedroom, office, living room and meeting room) and black for clothing [8]. Light wall colours might be preferred, because lighter wall and ceiling colours make rooms appear higher and more spacious to the perceiver [24–25].

Recently, Schloss and colleagues [11] investigated context-dependent colour preferences using a pre-selected colour sample. Participants viewed eight hues, each at two levels of saturation and at two levels of lightness in addition to five achromatic colours (black, white, and three shades of grey). They had to rate each colour on a preference scale for different contexts, both imagined and depicted (e.g. car, t-shirt, walls, sofa etc.), and for general preference (i.e. context-free patches). The authors observed that people preferred more saturated colours in general when compared to preferred colours for specific objects. They also preferred darker colours for objects (e.g. t-shirts, scarfs and couches) when compared to participants’ general preferences. Colour preferences for walls and trims deviated from this observation, i.e. the preferred colours were lighter than the preferences for colours in general. The authors concluded that differences in colour preferences in general, for walls and t-shirts could be mainly accounted for by lightness and saturation, and not by hue. To our knowledge, this is the only recent study that investigated colour preferences across contexts in a controlled and systematic way.

Different theories or models explain colour preferences as arising from biological-evolutionary, affective or ecological processes. Hurlbert and Ling [20] proposed that colour preferences are based on hard-wired cone opponent mechanisms in the human visual system that arose from evolutionary selection. Ou and colleagues [16, 26] empirically modelled colour preferences through association ratings of colours with affective word-pairs. The authors did this by matching colour-appearance dimensions (i.e. hue, lightness, and chroma) to affective dimensions derived from the ratings (warm-cool, heavy-light, active-passive, and hard-soft). Differences in colour preferences between cultures were explained by different colour-emotion associations, i.e. liked colours correlated with the affective dimensions fresh, clean and modern for Chinese participants but not for British participants. Finally, according to the ecological valence theory [15] colour preferences stem from colour-relevant experiences during an individual’s life. Positive emotional experiences with coloured objects lead to higher preferences of that colour (e.g. blue with clear sky) while negative emotional experiences lead to lower preferences of that colour (e.g. brown with rotten food). The authors reported that ecological valence theory had high explanatory power; the algorithm derived from the theory predicted 60% of the colour preferences [13]. The ecological valence theory mainly provides examples about object-based emotional experiences but the authors acknowledge that some experiences might be more abstract [13], for example that Chinese prefer red because it is a symbol of good luck. Thus, while evolutionary-biological accounts predict universal and sex-specific patterns in colour preference, accounts based on associative learning suggest that preferences arise from individual, colour-relevant experiences with objects or concepts. The role of conceptual associations in colour preferences remains however unexplored.

Of interest to our study, all the theories described above would make predictions regarding both liked *and* disliked colours, but disliked colours have received little attention so far. In some studies, participants see an entire colour sample and select the colour they like the most. The least often chosen colours are assumed to be the least preferred (i.e. yellow-green, black and white [27]). In a forced choice, paired-comparison task, participants choose the colour they like *more than* another colour presented simultaneously (e.g. [18, 20, 28]). As such, Hurlbert and Ling [20] found that yellow-green colours were among the least often chosen colours using this approach. A one-dimensional preference scale is then computed from, often lengthy, repetitions of all possible combinations of colour samples. The least chosen colour is assumed to represent the most disliked colour even though participants did not make this judgment directly. Especially situations with limited colour samples may not capture the extremes on this supposed unipolar dimension. In a variation of the paired-comparisons task, Simmons [29] first identified the 10 most unpleasant colours in a pilot study (dark and low in colourfulness colours) and then showed all possible combinations of any three colours to participants, asking them to pick the most unpleasant colour for each trial. Green-brown and yellow-brown were chosen most often as unpleasant colours in that study.

Alternative to the n-alternative forced choice task, aesthetic ratings are often used to assess colour preferences (e.g. [30–31]). In this approach, participants are asked to rate how much they like each of the sampled colours on a discrete or continuous scale (e.g. from *not at all* to *very much*). In a variation to this approach, Palmer and colleagues [13] presented their full set of colour stimuli prior to the rating task to anchor the response scale. The authors asked participants to give the highest and lowest ratings to their most and least preferred colours of the given sample, respectively. Following this approach, Palmer and Schloss [30] found that orange, yellow, and yellow-green colours were the least preferred colours. The ratings for these colours were especially low when they were dark and desaturated. The results from these studies as well as the previously mentioned reports together indicate that disliked colours seem to lie in the range of dull and dark yellows and greens. However, these conclusions are based on relative differences constrained by the available response range while disliked colours are not analysed specifically (for a notable exception, see [29]) and may potentially differ from overt preferences.

Taking the above literature into account, four observations are striking. Most studies 1) focus on hue (neglecting the role of brightness and colourfulness; e.g. [4, 7]), 2) force participants to choose from pre-selected colours rather than from an unrestricted colour range (e.g. [3, 15]), 3) decontextualize colour preferences and assume that general colour preferences, at least for hues, are generalizable to all contexts [13] and 4) focus on what people are attracted to (preferred, liked colours) and leave largely unattended what people avoid (disliked colours; e.g. [27]). If we want to advance our understanding of real-life colour preferences and their underpinnings, we consider it key to account for all four observations.

In the current study, we investigated how the three colour attributes as measured by hue, chroma (referring to the colourfulness attribute) and lightness (referring to the brightness attribute) varied between most and least preferred colours as a function of different contexts (general preferences and preferences for two imagined objects: interior walls and a t-shirt). Participants used a high-precision computerised colour picker to spontaneously choose any colour the monitor could display (i.e. unrestricted within the hardware constraints). This type of user production task [13] has the advantage of efficiently assessing preferences over a wide range of colours, without experimenters having to pre-determine a supposedly relevant sample. Our aim was to finely differentiate most and least preferred colours and test whether preference patterns differed between contexts. We further asked participants whether they could report any object or concept associated to the chosen colour and, if so, to provide a valence rating of that object or concept. Thus, we were able to test whether people would share previously reported colour preferences, i.e. that blue hues would be most preferred and yellow hues would be least preferred and that lighter and more chromatic (colourful) colours would be preferred to darker and less chromatic colours in general. Furthermore, we hypothesised that object context would influence colour preference judgments explaining a large amount of variability in colour preferences. Based on Schloss et al.’s [11] report, we expected that the level of lightness and chroma would differentiate object context conditions better than hues. More specifically, we expected that i) more chromatic colours would be chosen for generally preferred colours as compared to colour preferences for walls or a t-shirt and ii) lighter and less chromatic colours would be preferred for walls when compared to colours preferred in general or for a t-shirt. We also expected dark colours to be preferred for t-shirts. As for the associations, we expected to find valenced object-based associations for both most and least preferred colours, as shown before [15]. In addition, we explored potential concept-based associations to colour choices.

## Method

### Participants

Eighty-eight undergraduate students from the University of Lausanne, Switzerland (25 males) participated voluntarily in return for course credit. Their mean age was 20.97 years (*SD* = 3.303 years, range 18–39 years). Their vision was normal or corrected to normal for all participants. Only one male participant did not have normal colour vision according to the Ishihara’s Colour Blindness Test [32]. He was excluded from the analyses. We further excluded the data from two participants (a male and a female) because they seemed to have misunderstood the task: One returned an incomplete questionnaire and another chose most preferred colours in general for all six questions. We analysed the data of the final sample of 85 participants (23 males).

### Colour picker program

A computerized colour picker was developed by one of the co-authors (PS). The tool is similar to a colour production tool available online (http://www.limov.com/colour/navigate.lml). Common to this approach is a structured representation of colour patches showing variations relative to a centrally presented target colour patch. The colour selection process is sequential so that the target colour increases or decreases along various colour dimensions by clicking on the corresponding variations.

**Fig 1:**
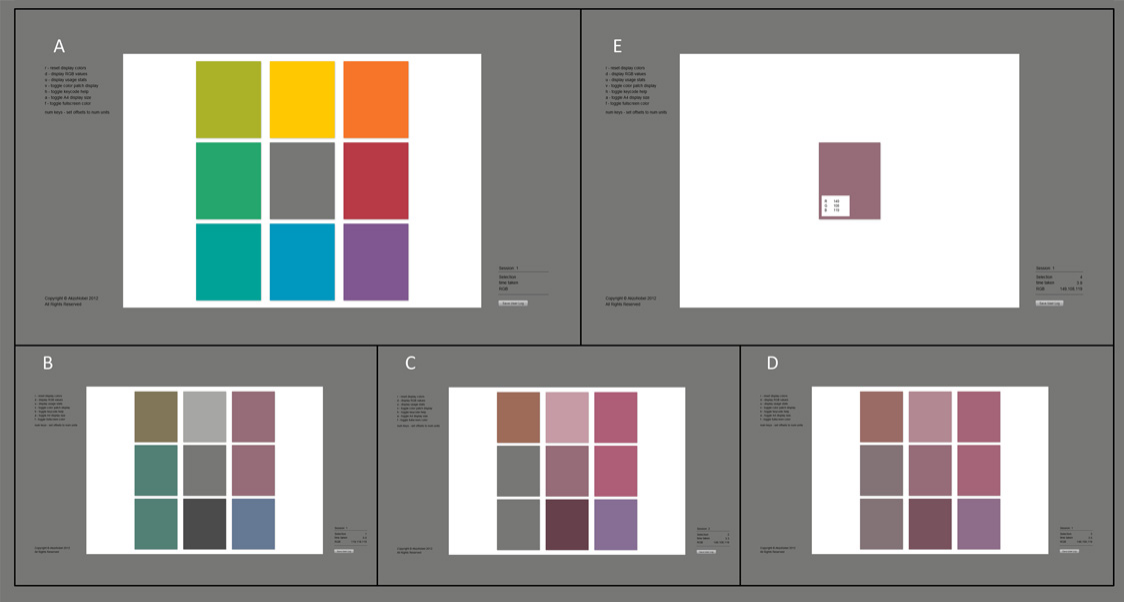
Illustration of the colour picker. Picture A refers to the starting screen and picture E refers to the screen of the final colour choice. Pictures B, C and D refer to possible intermediate selection steps of the program.

In our adaptation to this approach, nine square colour patches (red, orange, yellow, yellow-green, green, green-blue, blue, purple and grey) are presented on a white background rectangle on a mid grey background screen (Fig 1A). We provide in S1 Appendix the RGB colour values of the starting screen (Fig 1A) and the corresponding CIE xyY chromaticity values (measured with a Konica Minolta CS-100A chroma meter), which vary according to the monitor used. When presented with the starting screen, the participant is instructed to click on the patch that resembles the hue family of their most or least preferred colour (see Fig 1A). The selected colour patch becomes the centre, or reference patch, on the following frame (e.g. grey, Fig 1B). Variations of increased or decreased hue (yellow vs. blue and red vs. green), lightness, and chroma (related to, but not defined a priori as CIE L, C, a, b) are depicted as patches around the centre patch (Fig 1B–1D). At all times the participant can change the colour of the centre patch by clicking on the outer corner patches (depicting variations in hue), the upper or lower middle patch (depicting more or less lightness), and the right or left middle patch (depicting more or less chroma). The selected outer patch becomes the centre patch in the next frame. The difference of these outer patches from the centre patch decreases each time the centre patch is clicked (Fig 1). When the difference between the patches becomes too small to be rendered reliably, the outer patches disappear and the centre patch is proposed as the final colour choice (Fig 1E). At this stage the participant still has the option to correct their colour choice (if desired) by clicking on the centre patch once more, which brings back the outer patches. The participant can then further change the centre colour according to the above-defined procedure.

The colour picker program was designed as an intuitive colour production tool to visually navigate colour dimensions on any computer without a built-in calibration procedure to define colour choices in terms of a device-independent colour model. Thus, colour choices were recorded as RGB values, allowing the user to convert to other colour models taking into account laboratory specific conditions (monitor settings, ambient lighting, observer distance). Further output provided by the program was time (in seconds) taken and the number of clicks (steps) until reaching the final colour selection.

### Procedure

Participants first read and signed an informed consent form providing information about the study and their rights as participants in accordance with the guidelines of the Helsinki Declaration. This form explicitly stated that participants could opt out of the study at any time and that their responses to the experimental task were anonymous. Swiss Law does not require further ethical confirmation for this type of study so it was not evaluated by an institutional review board. We collected identifying and demographic information (name, sex, date of birth, native language, e-mail address) on a separate sheet in order to give course credit. The identifying information was stored separately from the data. We assessed colour-blindness with the 38 plates edition of the Ishihara’s Colour Blindness Test [32]. Participants then read the instructions of how to use the colour picker program. Afterwards, they were accompanied to an experimental room, only lit by a computer monitor. The colour picker program was run with Adobe Flash on Windows 7 Enterprise 2009 and viewed on Colour Edge CG243W 24.1" Widescreen LCD displays. Temperature of the monitors was set to 6500 K, gamma: 2.2, contrast: 100%, and brightness: 25%. Resolution was 1920 x 1200 pixels and the frame rate was 59.90 Hz. Eye-screen distance was approximately 70 cm. The patches were covering a viewing angle of approximately 16° x 17.85° and the final patch appeared at the viewing angle of about 4.90° x 5.73°.

Participants were asked to make six colour choices: the most and least preferred colours in three contexts: 1) in general, 2) for imagined interior walls of the room they would like to live in, and 3) for an imagined t-shirt they would like to wear. The context order was counterbalanced making four context presentation conditions: general, t-shirt, walls; general, walls, t-shirt; walls, t-shirt, general; t-shirt, walls, general. In this way, general preferences appeared either before or after the context-specific preferences (walls and a t-shirt). Participants chose the most preferred colour always before the least preferred colour.

After each colour choice, we probed participants for possible reasons for choosing that particular colour. That is, participants had to indicate one of the three options: a) whether they chose the colour because it reminded them of an object they know or have that has that colour, b) because it reminded them of a concept or c) that they simply (dis)liked the colour without a particular reason. If one of the two former options was selected, participants freely specified the object or the concept they associated to the chosen colour and used a linear scale to indicate how pleasant that object or concept was to them. They drew a line on the scale, which ranged from *very unpleasant* on the left to *very pleasant* on the right. Their responses were then converted into numeric expressions, which had a range from -45 (very unpleasant) to 45 (very pleasant), with the middle position representing a “neutral” point.

The experiment took around 20 minutes to complete, after which participants were thanked and fully debriefed.

### Colour conversion

As outlined in the colour picker program description above, we did not define all possible colours a priori within a device-independent colour model. Our aim was to evaluate differences in colour choices along colour dimensions modelling human colour perception. We thus measured the exact luminance (CIE Y in units per cd/m^2^) and chromaticity (CIE xy) of each final colour choice under the exact same laboratory testing conditions (monitor, lighting, observer distance) using a Konica Minolta CS-100A chroma meter. We converted these CIE xyY values into their corresponding XYZ tristimulus values. These were further converted into CIE LCh values, a cylindrical representation of CIE Lab colour space. We used the white background against which the colour patches were presented as the reference white (measured for each monitor separately, for exact values see S1 Appendix “colour 10”). We implemented colour-matching formulae described in Hunt & Pointer [6] in the statistical software R [33] package “colorscience” (see conversions “xyY2XYZ” on p. 133, “XYZ2Lab” on p. 136, and “Lab2LCHab” on p. 64 of [34]).

### Data analysis

**Fig 2:**
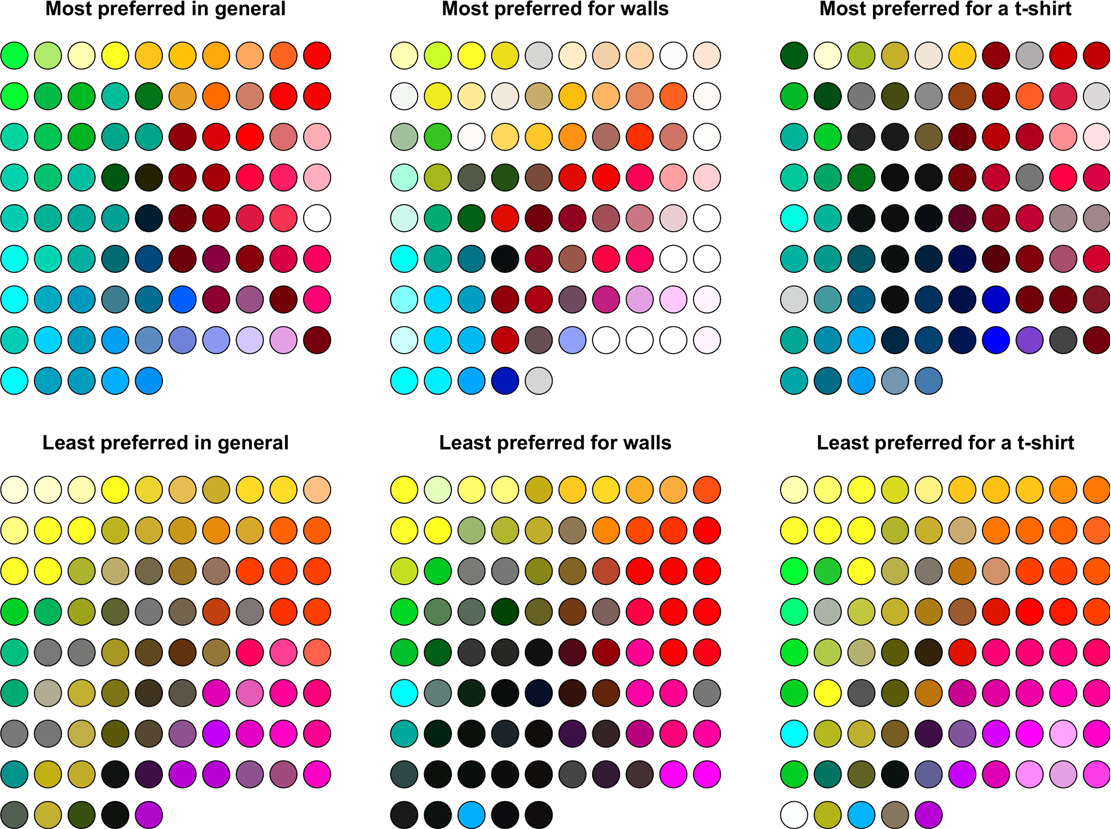
Most preferred and least preferred colour choices in three context conditions. Each colour patch represents a colour choice of one participant. Their positions are defined by 1) hue so that neighbouring hues on the colour circle (e.g. orange and yellow) appear next to each other in the graph, and 2) lightness so that darker colours appear closer to the centre as compared to lighter colours. The colours were derived from the CIE LCh values.

We performed statistical analyses on these CIE LCh values. The hue (h) parameter had a range of 0°-360°, chroma (C) had a range of 0–141, and lightness (L) had a range of 0–100. In order to analyse hue in perceptually relevant ways, we binned hue angles into categories [35–36]. The exact correspondence between ranges of hue angles and perceptually relevant hue categories (colour labels as one would use in everyday life) is not well established. Data driven propositions have recently been made in colour industry [35] and vision science (e.g. [36]). In the latter study [36], Parraga and Akbarinia conducted a psychophysiological experiment in which participants had to adjust a target colour patch until they found the colour that is “midway” between two colour words written at the bottom of the screen (e.g. “red” and “orange”). The results provided information on the boundaries of common colour categories such as between red and orange, orange and yellow etc., within the CIE Lab colour model. Of interest here, common colour labels mainly refer to hue, but some labels refer to darker or lighter variations of a hue. (e.g. dark yellow and dark orange are both referred to as “brown”, light red is referred to as “pink”; see Pane 81 vs. Pane 58 in Fig 2. of [36]). In our study, we focused on hue-specific categories not differentiating for instance between pink and red or yellow and brown. In sum, we took the correspondence values in [35] and the correspondence values depicted in Fig 2. of [35] to define nine hue categories (see Table 1). We included a separate category of achromatic colours (i.e. greys, from white to black). The respective cut-off values (i.e. chroma < 5) are illustrated in S1 Fig).

**Table 1:** CIE LCh hue categorization.

Colour	Focal hue	Hue range	Lightness	Chroma
Red	25°	346°-40°	any	> 5
Orange	57°	40°-72°	any	> 5
Yellow	87°	72°-105°	any	> 5
Yellow-Green	116°	105°-130°	any	> 5
Green	144°	130°-166°	any	> 5
Green-Blue	194°	166°-220°	any	> 5
Blue	244°	220°-275°	any	> 5
Purple	306°	275°-346°	any	> 5
Achromatic	none	any	any	≤ 5

Effects of hue were investigated using i) a marginal model for correlated multinomial responses, ii) multilevel logistic regressions, and iii) bootstrapping techniques (see results section for more details). We computed 2 (type of preference: most or least preferred) X 3 (context: general, walls, t-shirt) repeated measures ANOVA on lightness and chroma values, as well as on the time and number of clicks. Post-hoc comparisons were performed using Bonferroni tests controlling for multiple comparisons. All statistical tests were performed using statistical software R [33]. Results are based on two-tailed testing using an alpha level of 0.050.

## Results

### Preference Differences According to Hue, Lightness, and Chroma

Colour choices for each type of preference and context can be seen in Fig 2. A large inter-individual variation in colour choices can be noticed as well as striking variations between contexts. As we will outline in more detail below, there also seem to be general tendencies for colour choices. We visually present colour choices grouped by participant (S2 Fig) as well as distributed across the CIE LCh lightness and chroma dimensions (S1 Fig).

#### Hue

**Fig 3:**
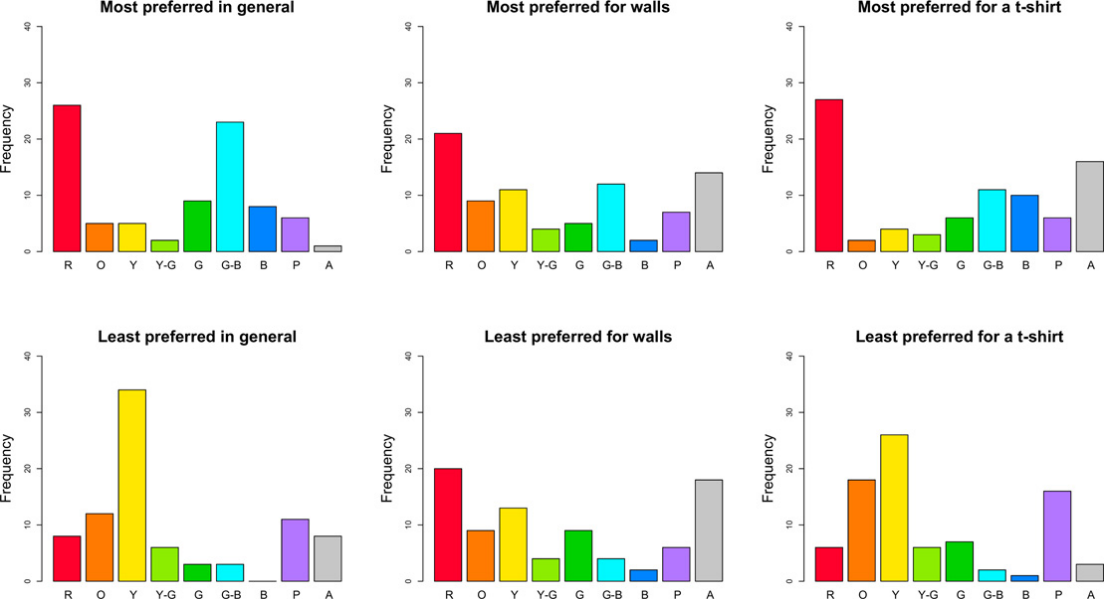
Distributions of colour choices according to hue for most and least preferred colours in three context conditions. This representation does not account for CIE LCh lightness and chroma values. R = red, O = orange, Y = yellow, Y-G = yellow-green, G = green, G-B = green-blue, B = blue, P = purple, and A = achromatic.

Fig 3 depicts the frequency distributions of colour choices according to the nine hue categories (see definition of hue as a categorical variable under “Data analysis”). We tested whether the hue of the colour choice depended on the type of preference and on the context by analysing the marginal model for correlated multinomial responses [37–38]. The hue choices differed between most and least preferred colour choices across the three context conditions, *G*^*2*^(21) = 69.39, *p* < 0.001. Colour choices also depended on the context across the preference conditions, *G*^*2*^ (28) = 49.16, *p* = .008. Finally, there was an interaction between the type of preference and context conditions, *G*^*2*^(35) = 88.14, *p* < 0.001. For most preferred hues, there was a significant difference between the hue distributions for 1) general preferences and walls, *G*^*2*^(7) = 27.14, *p* < .001, 2) general preferences and a t-shirt, *G*^*2*^(7) = 21.84, *p* = .003, but not between walls and a t-shirt (*p* = .055). For least preferred hues, there was a significant difference between the hue distributions for 1) general preferences and walls, *G*^*2*^(7) = 31.74, *p* < .001, 2) walls and a t-shirt, *G*^*2*^(7) = 33.82, *p* < .001, and 3) no difference between general preferences and a t-shirt (*p* = .260). The differences were also significant between most and least preferred hues for general preferences, *G*^*2*^(7) = 54.33, *p* < .001, a t-shirt, *G*^*2*^(7) = 58.80, *p* < .001, but not for walls (*p* = .630).

To investigate which hues underlay the observed differences in the frequency distributions (Fig 3), we performed a multilevel logistic regression analysis [39–40] for each hue category. We used type of preference and context as fixed effects and participant as random intercept (to account for inter-individual variations as each participant made a colour choice in each condition). In these analyses, we had to combine green-blue and blue hue categories due to a low number of responses in some conditions. There were several main effects of type of preference: red, ^*2*^(1) = 14.37, *p* < .001, orange, ^*2*^(1) = 5.88, *p* = .015, yellow, ^*2*^(1) = 23.13, *p* < .001, green-blue to blue, ^*2*^(1) = 28.38, *p* < .001, and purple, ^*2*^(1) = 3.87, *p* = .049, hue categories. More concretely (see also Fig 3), red and green-blue to blue were chosen more often as most preferred hues across contexts. Yellow, orange and purple were chosen more often as least preferred hue across contexts. Interactions between type of preference and context were significant for red, ^*2*^(2) = 10.53, *p* = .005, orange, ^*2*^(2) = 7.12, *p* = .028, yellow, ^*2*^(2) = 13.02, *p* = .001, and the achromatic, ^*2*^(2) = 12.92, *p* = .002, categories (Fig 3). The remaining comparisons were not significant (all *ps* > .110). Red hue was frequently chosen in all context conditions, apart from least preferred colour in general and least preferred hue of a t-shirt (Fig 3). Orange and yellow hues were frequently chosen as least preferred hue in all context conditions, but were a relative frequent choice when it came to most preferred hues for walls (Fig 3). Finally, achromatic colours were frequent choices for most and least preferred colours for walls, and as most preferred colour for a t-shirt.

We finally tested whether certain hue categories were chosen more often than others within each condition using bootstrapping [41, 42]. This analysis enabled us to control for multiple comparisons (see Table 2). We randomly drew 100’000 samples from the initial hue frequency distribution (*N* = 85), and did so separately for each of the 3 x 2 conditions (with replacement of the sample drawn). On these bootstrapped samples, we determined the distribution of the differences in choice proportions between all possible hue pairs (p_i_—p_j_, i.e. 36 pairs in total), and calculated a confidence interval for each hue pair difference that corrects for multiple comparisons (alpha/(k*(k – 1))). Here, alpha corresponds to the significance threshold of .050 and k corresponds to the number of possible hue categories (here k = 9). Table 2 summarizes for each pairwise comparison whether the proportional difference is significantly different from zero using a letter-based representation [43].

**Table 2:** Bootstrapped pairwise comparisons.

Hue	General				Walls				T-shirt			
	Most		Least		Most		Least		Most		Least	
	*n (%)*	*Letter*	*n (%)*	*Letter*	*n (%)*	*Letter*	*n (%)*	*Letter*	*n (%)*	*Letter*	*n (%)*	*Letter*
**Red**	26 (30.6)	*a*	8 (9.4)	*ab*	21 (24.7)	*a*	20 (23.5)	*a*	27 (31.8)	*a*	6 (7.1)	*abc*
**Orange**	5 (5.9)	*b*	12 (14.1)	*a*	9 (10.6)	*abc*	9 (10.6)	*abc*	2 (2.4)	*b*	18 (21.2)	*ad*
**Yellow**	5 (5.9)	*b*	34 (40.0)	*c*	11 (12.9)	*abc*	13 (15.3)	*abc*	4 (4.7)	*bc*	26 (30.6)	*d*
**Yellow-Green**	2 (2.4)	*b*	6 (7.1)	*ab*	4 (4.7)	*bc*	4 (4.7)	*bc*	3 (3.5)	*bc*	6 (7.1)	*abc*
**Green**	9 (10.6)	*abc*	3 (3.5)	*ab*	5 (5.9)	*bc*	9 (10.6)	*abc*	6 (7.1)	*bc*	7 (8.2)	*abc*
**Green-Blue**	23 (27.1)	*ac*	3 (3.5)	*ab*	12 (14.1)	*abc*	4 (4.7)	*bc*	11 (12.9)	*abc*	2 (2.4)	*b*
**Blue**	8 (9.4)	*bc*	0 (0.0)	*b*	2 (2.4)	*b*	2 (2.4)	*b*	10 (11.8)	*abc*	1 (1.2)	*b*
**Purple**	6 (7.1)	*b*	11 (12.9)	*a*	7 (8.2)	*abc*	6 (7.1)	*abc*	6 (7.1)	*bc*	16 (18.8)	*acd*
**Achromatic**	1 (1.2)	*b*	8 (9.4)	*ab*	14 (16.5)	*ac*	18 (21.2)	*ac*	16 (18.8)	*ac*	3 (3.5)	*bc*
**Total**	85 (100)		85 (100)		85 (100)		85 (100)		85 (100)		85 (100)	

We compared the proportions of hue choices for most and least preferred colours in general, for walls and for a t-shirt. We show the number of participants choosing a particular hue (n), percentage of participants (from total N) making this selection (%). If the same letter is shown for at least two hues, the letter indicates that the pairwise comparison was not significant. In other words, the hues were equally often chosen for these context conditions. If two hues have no letter in common, the pairwise comparison was significant, i.e. the hues were not equally often chosen for these context conditions (each condition analysed separately). All p–values were controlled for multiple comparisons using bootstrapping, i.e. the significance alpha level was set at ≤ .0006.

Based on these bootstrapped pairwise comparisons (see Table 2, see also Fig 3), red and green-blue seemed to be very liked hues in general, chosen by 57.6% of all participants, followed by green and blue hues, which on top of that accounted for 20% of choices. Yellow hue was chosen as the least preferred hue by 40% of all participants, a proportion that strongly differed from those of all other hues. Also orange and purple were disliked by 27.1% of the participants. When comparing most preferred hues for interior walls, red was chosen by the highest number of participants (24.7%), which was closely followed by choices of achromatic hues, green-blue, yellow, orange, and purple (in decreasing order or proportion size). However, a strikingly similar distribution of hue choices was observed for least preferred hues for walls. When comparing hues chosen for a t-shirt, red was clearly the most often preferred hue and accounted for 31.8% of choices. Next came achromatic colours and green-blue and blue hues, chosen by 43.5% of all participants. As for least preferred hues for a t-shirt, yellow was most often disliked (30.6% of all participants) as well as orange (21.2% of all participants) and purple (18.8% of all participants).

#### Lightness

**Fig 4:**
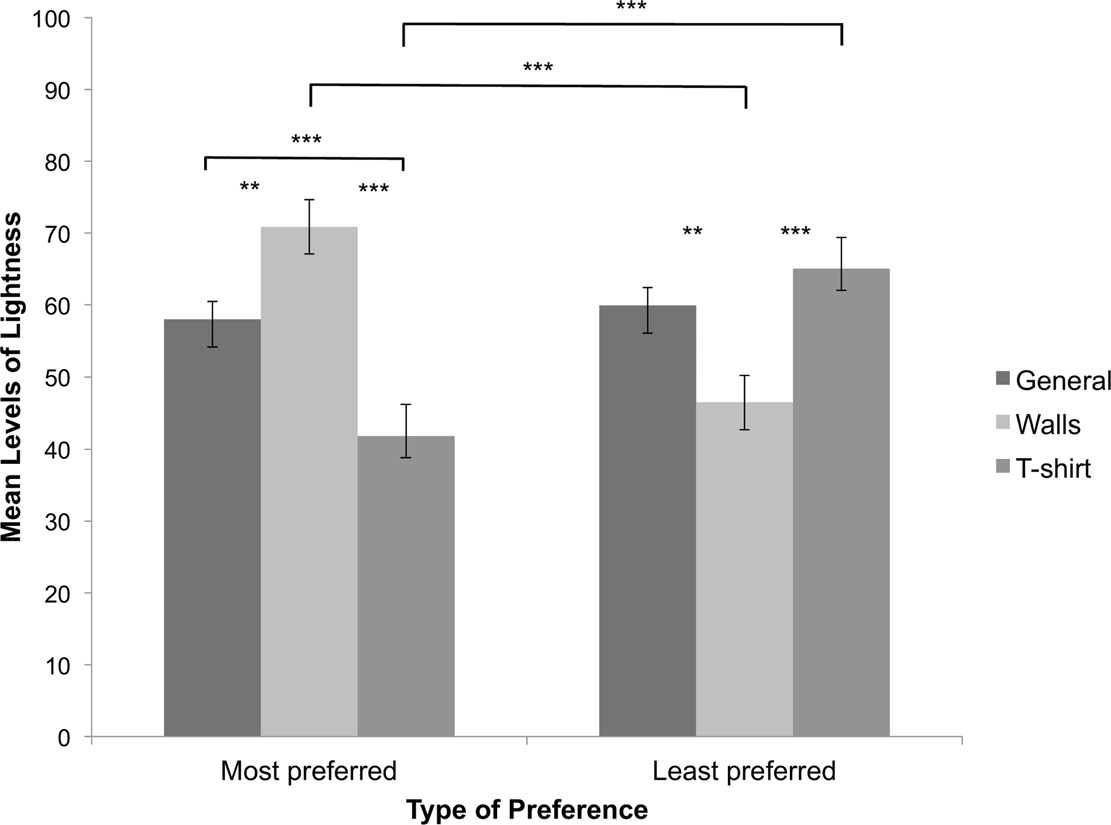
Lightness levels of colour choices. Depicted are the mean lightness levels for the three context conditions and most and least preferred colours, separately. Bars indicate one standard error of the mean. We indicate significant pairwise comparisons (** p < 0.*010*, *** *p < 0*.*001)*.

The repeated measures ANOVA on CIE LCh lightness values showed no main effect of type of preference, *F*(1, 84) = 0.011, *p* = .917, partial *η*^2^ < .001 (see Fig 4). There was however a significant main effect of type of context, *F*(2, 168) = 3.784, *p* = .025, partial *η*^2^ = .043, ε = .904. The colours for a t-shirt were darker than colours for general preferences (*p* = .018). The remaining pairwise comparisons were not significant (*ps* > .139; see Fig 4). The interaction between type of preference and context was also significant, *F*(2, 168) = 40.363, *p* < 0.001, partial *η*^2^ = .325. Post-hoc comparisons showed that for walls the most preferred colours were lighter than the least preferred colours (*p* < .001). The difference for a t-shirt was in the opposite direction: the most preferred colours were darker than the least preferred colours (*p* < .001). The pairwise comparison between most and least preferred colours in general was not significant (*p* = .938; see Fig 4). When comparing most preferred colours between contexts, colours were lighter for general preferences than for a t-shirt (p < .001). Colours were lighter for walls than for general preferences (p = .001) and a t-shirt (p < .001), respectively. When comparing least preferred colours between contexts, colours were darker for walls than for a t-shirt (p < .001) and general preferences (p = .001), respectively. Colours did not differ in lightness for least preferred colours in general and those for a t-shirt (p = 0.191; see Fig 4).

#### Chroma

**Fig 5:**
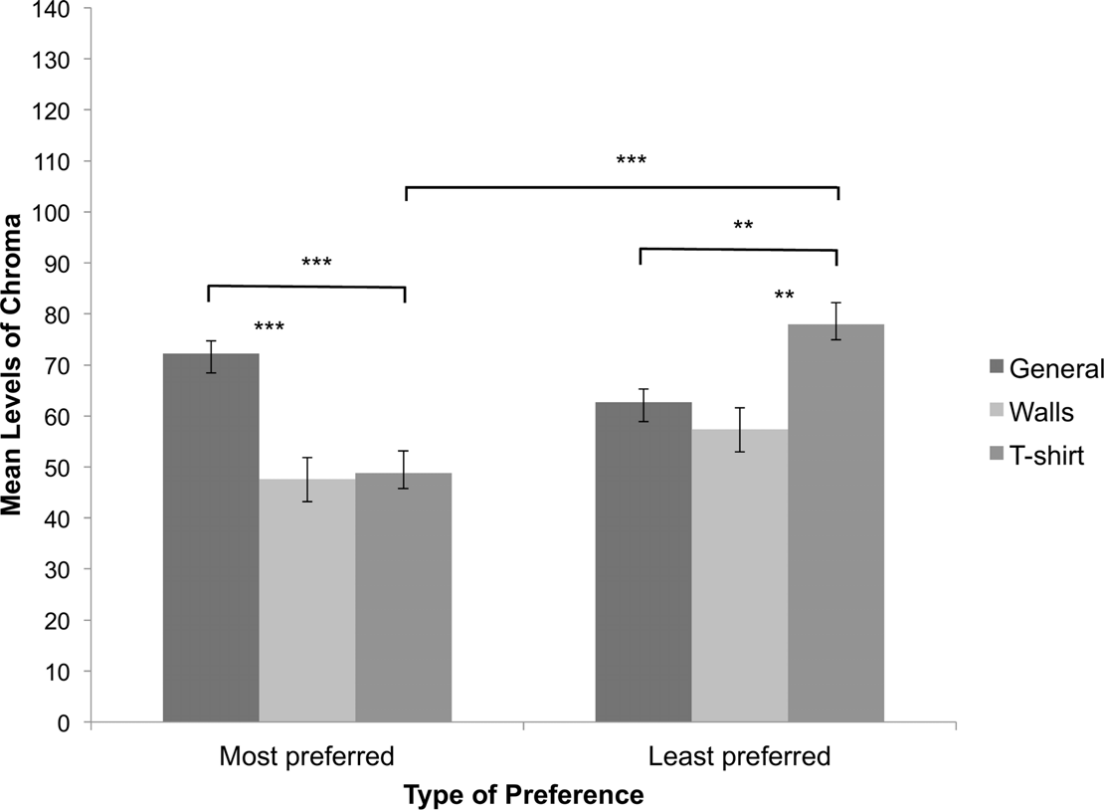
Chroma levels of colour choices. Depicted are the mean chroma levels for the three context conditions and most and least preferred colours, separately. Bars indicate one standard error of the mean. We indicate significant pairwise comparisons (** p < 0.*010*, *** *p < 0*.*001)*.

The repeated measures ANOVA showed a significant main effect of type of preference, *F*(1, 84) = 5.337, *p* = .023, partial *η*^2^ = .060 (see Fig 5). The least preferred colours were more chromatic than the most preferred colours. We also found a significant main effect of context, *F*(2, 168) = 9.060, *p* < .001, partial *η*^2^ = .097, ε = .924. The chroma levels were less high for walls as compared to general preferences (*p* = .001) and a t-shirt (*p* = .017), respectively. The chroma levels were comparable for general preferences and a t-shirt (*p* = .555). The interaction between type of preference and context was significant, *F*(2, 168) = 13.000, *p* < 0.001, partial *η*^2^ = .134, ε = .868. Post-hoc tests showed that most preferred colours for a t-shirt were less chromatic than least preferred colours for a t-shirt (*p* < .001). Analogue comparisons for general preferences *(p* = .081) and for walls (*p* = .149), respectively, were not significant (see Fig 5). When comparing chroma levels for most preferred colours between context conditions, we found that participants chose more chromatic colours for general preferences than walls (*p* < .001) and a t-shirt (*p* < .001). There were no chroma differences for walls and a t-shirt (*p* = 1.000). Analogue comparisons for least preferred colours showed that participants chose less chromatic colours for a t-shirt as compared to general preferences (*p* = .003) and walls (*p* = .004). There were no chroma difference between general preferences and preferences for walls (*p* = 1.000; see Fig 5).

### Differences in the Selection Process of Most and Least Preferred Colours

Here, we analysed how most and least preferred colour choices differed between each other on two types of variables: duration of the selection process (measured in seconds and number of clicks) and the number and type of valenced associations indicated for the selected colour.

#### Duration of the selection process

The repeated measures ANOVAs on the time taken and the number of clicks showed significant main effects of type of preference for the number of clicks, *F*(1, 84) = 6.922, *p* = .010, partial *η*^2^ = .076, and total time, *F*(1, 84) = 12.990, *p* = .001, partial *η*^2^ = .134. It took more clicks and more time to arrive at the most preferred colours than least preferred colours. There was a significant main effect of context for number of clicks, *F*(2, 168) = 3.102, *p* = .048, partial *η*^2^ = .036. Participants made more clicks to select colours for walls compared to a t-shirt (*p* = .031). The remaining pairwise comparisons were not significant (*ps* > .668). There was no significant main effect of context for total selection time, *F*(2, 168) = 1.568, *p* = .212, partial *η*^2^ = .018. There were further no significant interactions between type of preference and context condition neither for number of clicks, *F*(2, 168) = .798, *p =* .452, partial *η*^2^ = .009, nor for total selection time, *F*(1, 168) = 2.257, *p* = .108, partial *η*^2^ = .026.

#### Personal valenced associations and pleasantness ratings

We asked participants whether they selected a particular colour as most or least preferred because i) it reminded them of an object they know or own that has that colour; ii) it reminded them of a concept; or iii) they had no reason. Examples of frequently listed object-based associations include *plants*, *t*-*shirts*, and *sun* (most preferred); *Barbie* (least preferred). Examples of concept-based associations include *tranquillity*, *liberty*, and *elegance* (most preferred); *sadness*, *aggression*, *pessimism (*least preferred).

**Fig 6:**
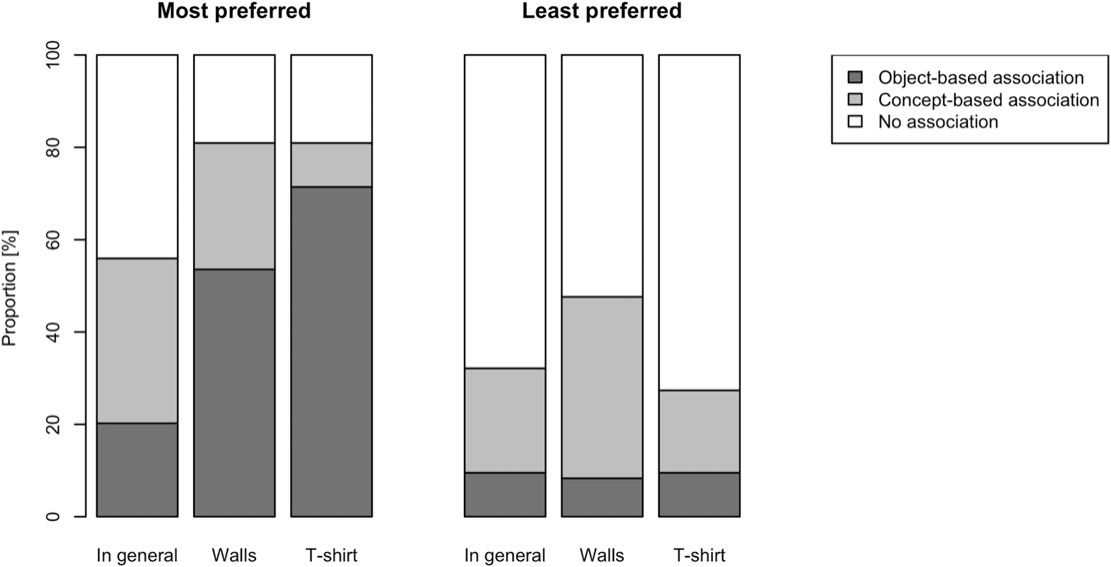
Cumulative bar chart of reported associations to colour choices. The portions of the cumulative bars indicate the percentage of the participants associating their chosen colours with an object, a concept, or nothing (i.e. no association). Colour choices are split by type of preference and context condition.

To analyse the distribution of associations (object- or concept-based, or no association) we first counted the number of associations that each participant made for most and least preferred colours, without separating the context conditions. Stuart-Maxwell [44] test, which accounts for repeated measurements, showed that the distribution was significantly different for most as compared to least preferred colours, χ^2^(df = 2, n = 84) = 40.8, *p* < .001. Participants reported more associations (object or concept) for most preferred colours than least preferred colours (Fig 6). This implies that participants reported no associations more frequently for least preferred colours than for most preferred colours (Fig 6). The follow-up Stuart-Maxwell tests indicated that the tendency to report more associations for most preferred colours compared to least preferred colours was true for each context condition separately: general, χ^2^(df = 2, n = 84) = 9.13, *p* = .010, walls, χ^2^(df = 2, n = 84) = 33.2, *p* < .001, and a t-shirt, χ^2^(df = 2, n = 84) = 48.9, *p* < .001.

When comparing the number of associations to objects versus concepts, the paired Wilcoxon signed rank tests showed that object-based associations were more frequently listed for most preferred colours (*V* = 1520.5, *Z* = 4.13, *p* < .001) while concept-based associations were more frequently listed for least preferred colours (*V* = 277.5, *Z* = -3.61, *p* < .001).

**Fig 7:**
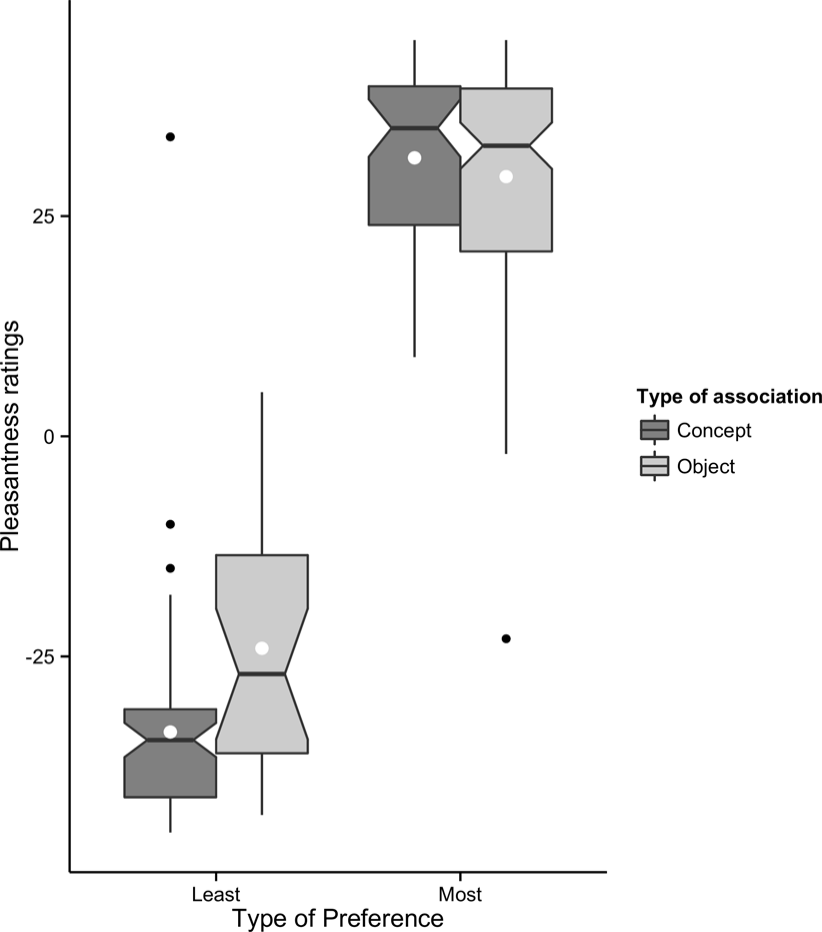
Pleasantness ratings of objects and concepts associated to selected colours. Notches indicate the confidence interval of 95% from the median (horizontal line within the box) and the white dot in the box indicates the mean. The edges of the boxes indicate the range of responses of 50% of participants. The whiskers indicate the range responses without extreme outliers, which are indicated as dots.

Fig 7 shows that pleasantness ratings of objects and concepts associated with most preferred colours were distributed in the positive range. Pleasantness ratings of objects and concepts associated with least preferred colours were distributed in the negative range. This meant that liked colours were often pleasant while disliked colours were often unpleasant, disregarding whether they were associated with objects or concepts.

## Discussion

In this study, we investigated context-specific colour preferences, namely for general colour preferences, for imagined interior walls and for an imagined t-shirt. We aimed to describe colour preferences beyond general hue preferences by equally considering chroma and lightness. We measured both ends of the preference spectrum (most and least) to test which factors (i.e. hue, chroma, and lightness) explained the observed differences between most and least preferred colours chosen in these contexts. We refrained from using pre-selected colours and opted for an unrestricted colour selection approach. We here developed a colour picker program that allowed a precise colour selection from the full range of colours that our computer screens could produce. We tested for differences in the selection behaviour between most and least preferred colours in terms of response times and reported associations.

Hue analyses showed that across contexts, red and blue to green-blue hues were more often liked than disliked, while orange, yellow, and purple hues were more often disliked than liked. Several hues (i.e. yellow-green, green and achromatic) were neither liked nor disliked across contexts. Hue preferences differed between contexts and between types of preference. Participants’ most preferred hues in general were red and green-blue, while orange, yellow and purple were most often chosen as least preferred hues in general. The overall hue profile of the colour choices for walls was less contrasting and especially similar between most and least preferred colours. This meant that hue could not differentiate well between most and least preferred colours for walls. Hue preferences for a t-shirt seemed to closely resemble general hue preferences, with red being favourite and orange, yellow and purple being least favourite hues. A wide variation of hue preferences suggests that context plays a major role in colour selection and that other colour parameters (i.e. chroma and lightness) are important.

In contrast to least preferred colours, most preferred colours were lighter for walls but darker for a t-shirt. Most preferred colours were less chromatic for a t-shirt than least preferred colours, while chroma did not differ between most and least preferred colours in general or for walls. Hence, hue, chroma and lightness accounted well for the observed differences between most and least preferred colours for certain context conditions but, importantly, the relationship between the three colour parameters and type of preference was different for different contexts.

### General Colour Preferences

Our results on general hue preferences replicated previous findings and provided some new insights into the literature. Similarly to previous scientific [7–11, 15, 45] and marketing [3, 46] studies, we observed that in general green-blue (sometimes referred to as “cyan”, “turquoise”, “teal”, etc.) was the most preferred hue. Usually, blue is reported to be the favourite hue [7–11]. Others, however, observed a high preference for green-blue [11, 13, 15]. Fortmann-Roe [21] reported that Twitter users often chose cyan as a theme colour of their account. We also replicated a general dislike for yellow [7–11]. Noteworthy, disliked yellows were often dark (and of low colourfulness), equally supporting previous findings that preferences for yellow abate with decreasing lightness levels [15]. According to Parraga and Akbarinia [36], people would usually refer to such “yellow” colours as “brown”, New to the literature, we found additional hues that were either most (red) or least (orange and purple) preferred. Red hue popularity might be explained by a large number of females in our sample. Hurlbert and Ling [20] reported a high female preference for reddish colours (but see [28]). Also, Zentner [47] reported that red is the third favourite colour of adults (after dark and bright blue). Yellow-green was not among the least preferred hues in our study, even though a number of previous studies identified chartreuse [15], green-brown [29] or yellow-green [48] as disliked or unpleasant colours. Hence, the current study describes a more diverse sample of most and least preferred hues and provides some refinement to the field.

We expected to replicate that most preferred colours in general would be lighter and more colourful than least preferred colours [13]. The expectations were neither confirmed for chroma, an attribute of colourfulness (which showed only a tendency towards significance), nor for lightness. For example, Schloss et al. [11] observed large differences in preference ratings between muted (i.e. colour of medium low colourfulness) and colourful colours, with colourful colours receiving higher ratings. These authors did not, however, observe any differences between preference ratings of dark, muted and light colours. Early studies often reported increased preference with increased lightness [14, 17]; however, these results were not replicated in recent studies [11, 15]. Palmer et al. [13] explained this through the fact that lightness is confounded with colourfulness. Since people generally prefer highly colourful colours and preference for different hues peak at different lightness levels (e.g. yellow at high lightness levels, blue at low lightness levels, red and green at medium lightness levels), preference for lightness also varies. Thus, in contrast to this previous report [11], our findings comparing the two extremes of the preference spectrum did not show an overall preference of colourfulness, indicating that least preferred colours can also be very chromatic if the person is allowed to choose the exact colour from an unrestricted colour range.

### Colour Preferences in Context

Even though general hue preferences are thought to generalise to specific objects ([11], but see [8]), we observed a more nuanced picture. Adopting an unrestricted colour selection tool as developed for the current study, we obtained a wide variation in most and least preferred colours compared to previous studies. For example, Schloss and colleagues [11] reported that blue hues received the highest preference ratings for all object context conditions (including general preferences, walls and t-shirts). In our study, despite red and green-blue being the most popular hues in general, people did not single out these hues for walls. Also, yellow was most frequently chosen as least preferred hue in all conditions. However, it was also a frequently chosen hue for most preferred wall colours. The latter finding corresponds with the observation that “magnolia” paint is very popular for interior walls [49]. Thus, results generally showed that hue preferences differed largely between context conditions and demonstrated that general hue preferences did not generalize to object-specific hue preferences.

Chroma and lightness values for most and least preferred colours were different for walls and for a t-shirt. As for hue, general preferences for chroma and lightness did not generalise across contexts. In line with previous studies [8, 11, 23], most preferred colours in general were lighter than those preferred for a t-shirt, but darker than those preferred for walls. Schloss and colleagues [11] reported that lighter colours received highest preference ratings for walls and darkest colours received highest preference ratings for a t-shirt. Lighter colours make interior spaces appear larger [24–25]. Hence, participants’ choices might have been influenced by perceptual biases and functionality as well as genuine preferences. Furthermore, the relevance of appropriateness for colour choices has been previously shown in emotional context [22]. In sum, this study shows that general colour preferences differ from contextual colour preferences. If indeed learned, valenced associations create at least part of our colour preferences [15], they are likely to be different for different contexts and therefore less likely to be biologically determined [20].

### Differences in Selection Processes of Most and Least Preferred Colours

As discussed in the introduction, studies on colour preferences tended to focus on liked colours while largely neglecting disliked colours. It is assumed that the same mechanisms that guide liked colours also guide disliked colours. For example, according to the ecological valence theory [15], liked colours are linked to positive experiences with same coloured objects and disliked colours are linked to negative experiences with same coloured objects. Indeed, we observed that objects associated with most preferred colours received highly pleasant ratings while objects associated with least preferred colours received highly unpleasant ratings. In addition, the same pattern occurred for associated concepts, empirically extending the supposed relationship between positive experiences and colour preferences [15]. Yet, participants listed more associations for most preferred colours compared to least preferred colours. When they did report an association for least preferred colours, it was more often concept-based as compared to most preferred colours, which had more object-based associations. To our knowledge, no existing theory explains the observed pattern (or rather lack) of associations to disliked colours. We also noticed that participants took less time and made fewer clicks to choose their least preferred colours compared to most preferred colours. On the basis of these results, we conjecture that the selection of least preferred colours is more immediate and effortless than the selection of most preferred colours, and less prone to associated valenced experiences. Strauss and colleagues [29] also documented that colour preferences did not decrease after repeated exposure to unpleasant items of that colour (e.g. red and blood), while they did increase after exposure to pleasant items of that colour (e.g. red and berries). The latter finding and this study add merit to the idea that processes involving different levels of cognitive deliberation may guide the selection and formation of most and least preferred colours.

### Measuring Colour Preferences

Two methodological innovations may contribute to future research on colour preferences. First, previous studies used a more or less limited range of colours to test colour preferences because of constrains that were imposed by measurement techniques. For example, in a forced-choice paired comparison task (e.g. [20, 29]), the number of colours that can be presented is limited by the time it will take to respond to all possible combinations of those colours. Pre-selection limits the number of hue, colourfulness, and brightness combinations that can be measured and findings are limited to the selected range. In this study, we adopted a colour production approach using a colour picker that allows the participant to intuitively navigate the colour dimensions and gradually narrow down the colour selection range to finally achieve the exact colour they wish to select. This method broadens the range of colours included in the analyses and allows the measurement of contributions of hue, colourfulness and brightness to individually chosen colour preferences. One limitation of the present colour picker is that the initial screen displays widely varying and highly colourful hues. This starting situation may have guided participants to initially select highly colourful colours, resulting in an attenuated likelihood to change hue in later stages. In our study, the colourfulness varied according to context (low colourfulness for walls) questioning that participants would be stuck with highly colourful colours.

A second innovation is the focus on both extremes of the colour preference dimension. That is, observations that certain colours receive higher ratings than others do not necessarily imply that the former colours are actually liked and would be chosen in a realistic choice setting. Looking at the graphs of hue ratings reported by Schloss and colleagues ([11], Fig 5), one can notice that ratings of the majority of colours cover close to a neutral mid-point of the preference scale and below. That is, although the preference scale ranges between -100 and +100; preference ratings for all contexts seem to fall between -75 (yellow low in colourfulness) and 35 (light blue). In an extreme case of colour preference rating for an imagined car type (sedan), only blue colours seem to receive ratings around a neutral mid-point and all other colours have ratings between -75 and 0. In such cases, it would be informative to statistically compare the average preference rating of each colour to the “neutral” point (i.e. neither liked or disliked) in order to draw more precise conclusions on the actual rather than relative preference. In our approach we are confident that participants’ choices reflect genuine liking or disliking because we instructed participants to choose colours they preferred the most and the least from an unrestricted sample (i.e. all the possible colours). Future studies using this approach could include multiple choices at each scale end or target choices at the mid-point, i.e. colours to which people are indifferent. This methodological approach could be useful when studying stability of colour preferences over time or investigating synaesthesia, for example.

## Conclusions

The current study offers methodological innovations in the study of colour preferences, from which three main conclusions can be drawn to guide future research. First, using an unrestricted colour range we demonstrated that colour preferences should be measured as a combination of hue, colourfulness and brightness since all three parameters distinguish well most and least preferred colours. Second, we showed that general colour preferences do not generalise to context specific colour preferences. Colour preferences should be studied in context when the interest lies in colour preferences for a specific object. Third, future research should focus more on disliked colours because they seem to be processed differently from liked colours. We hope that future research will benefit from the methodological innovations presented here and launch systematic investigation of colour preferences in context, which in turn would help to incorporate new observations into existing theories.

## Supporting Information

S1 Appendix The colour parameters (RGB and xyY) of the colours displayed on the starting screen of the colour picker program. (PDF)

S1 Fig A two-dimensional (chroma x lightness) distribution of most and least preferred colours for the three context conditions. The red line indicates the cut-off point for achromatic colours.
(TIF)

S2 Fig Colour choices for context and preference condition grouped by participant. One large rectangle represents one participant. Top line of three squares in each rectangle represents most preferred colours while bottom line of three squares represents least preferred colours. Small squares represent a colour choice for each context (columns left to right: general, walls, t-shirt).
(TIF)
