# Supplementary material for: Most and Least Preferred Colours Differ According to Object Context: New Insights from an Unrestricted Colour Range
Source: PLoS One. 2016 Mar 29;11(3):e0152194. doi: 10.1371/journal.pone.0152194 (PMC4811414; doi:10.1371/journal.pone.0152194)
Supplement: S1 Appendix — (PDF) [file pone.0152194.s001.pdf]

S1 Appendix: The colour parameters of the initial colours displayed by the colour picker program. The numbers of the colours match to the patches of the initial screen of the colour picker – see below.

Colour		All monitors			Monitor A			Monitor B			Monitor C		
		R	*G*	*B*	*Y*	*x*	*y*	*Y*	*x*	*y*	*Y*	*x*	*y*
1.	Yellow-Green	171	178	40	48.3	.406	.504	49.9	.390	.514	43.1	.404	.504
2.	Yellow	255	200	0	77.0	.485	.458	78.6	.468	.469	69.3	.482	.458
3.	Orange	246	117	40	41.9	.583	.371	42.0	.573	.378	38.4	.580	.372
4.	Green	36	166	109	30.4	.199	.489	32.5	.198	.495	28.2	.198	.487
5.	Grey	119	119	118	20.4	.324	.348	20.9	.313	.353	19.2	.323	.346
6.	Red	183	58	70	17.4	.578	.309	16.7	.569	.310	16.2	.575	.309
7.	Blue-Green	0	162	151	27.2	.176	.386	28.9	.177	.391	25.9	.175	.359
8.	Blue	0	152	190	25.1	.166	.286	26.8	.166	.292	23.4	.165	.283
9.	Purple	128	87	144	14.2	.330	.242	14.3	.319	.242	13.6	.322	.237
10.	White	255	255	255	112	.323	.345	116	.314	.352	105	.321	.344

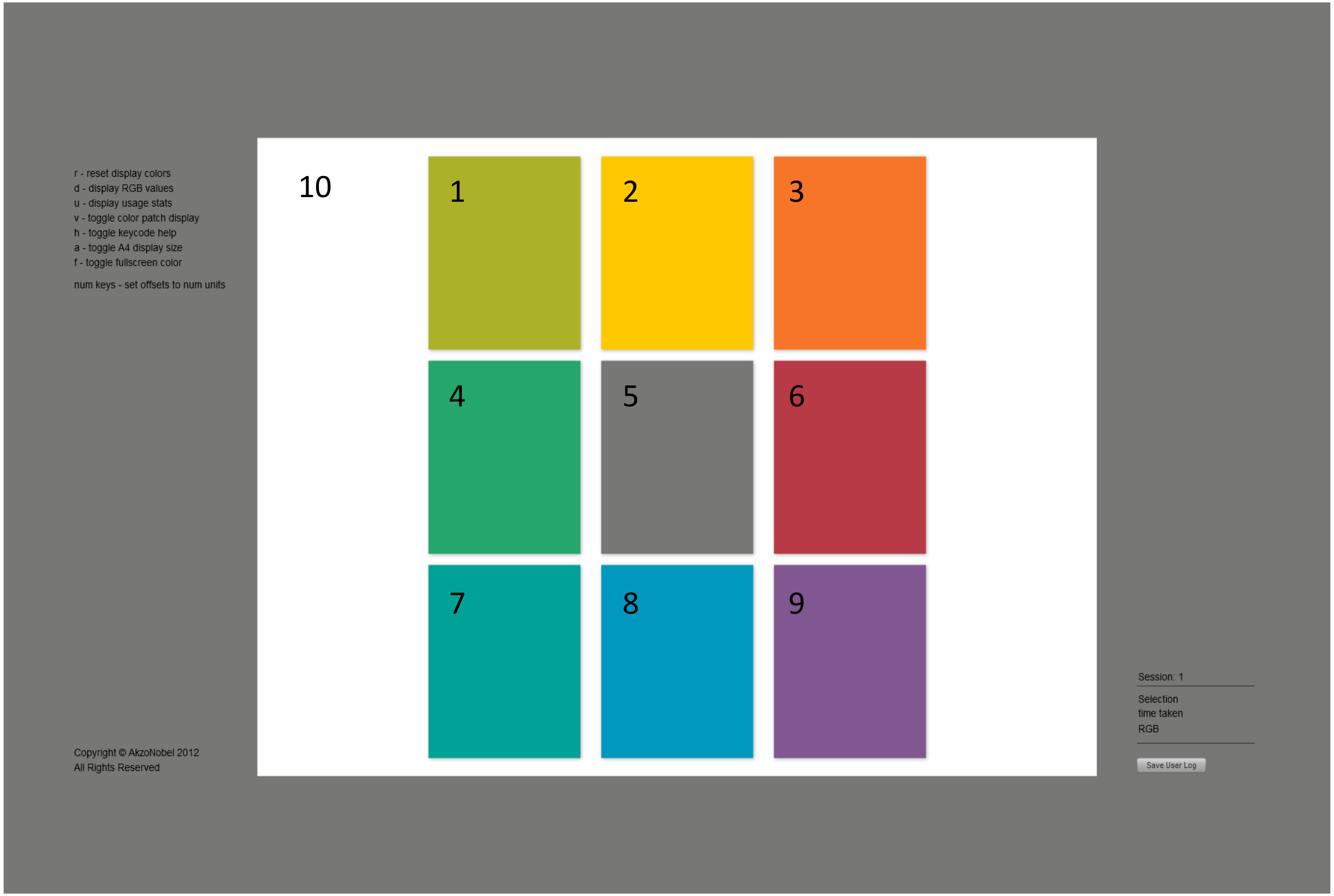
r - reset display colors
d - display RGB values
u - display usage stats
v - toggle color patch display
h - toggle keycode help
a - toggle A4 display size
f - toggle fullscreen color
num keys - set offsets to num units
10
1
2
3
4
5
6
7
8
9
Session: 1
Selection
time taken
RGB
Save User Log
Copyright © AkzoNobel 2012
All Rights Reserved
