# Supplementary figures and images for: Most and Least Preferred Colours Differ According to Object Context: New Insights from an Unrestricted Colour Range
Source: PLoS One. 2016 Mar 29;11(3):e0152194. doi: 10.1371/journal.pone.0152194 (PMC4811414; doi:10.1371/journal.pone.0152194)

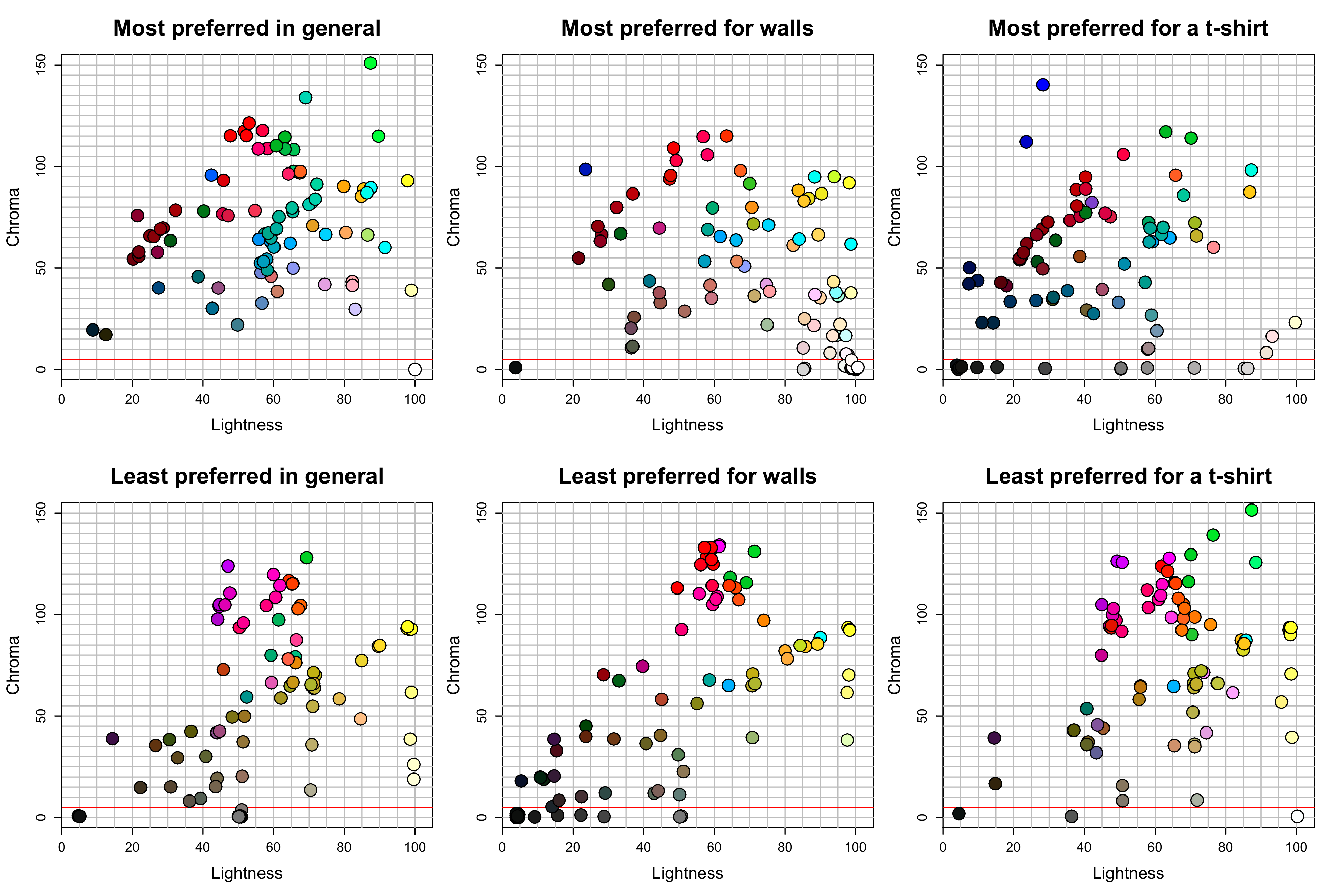

Supplement: S1 Fig — The red line indicates the cut-off point for achromatic colours. (TIF) [file pone.0152194.s002.tif]

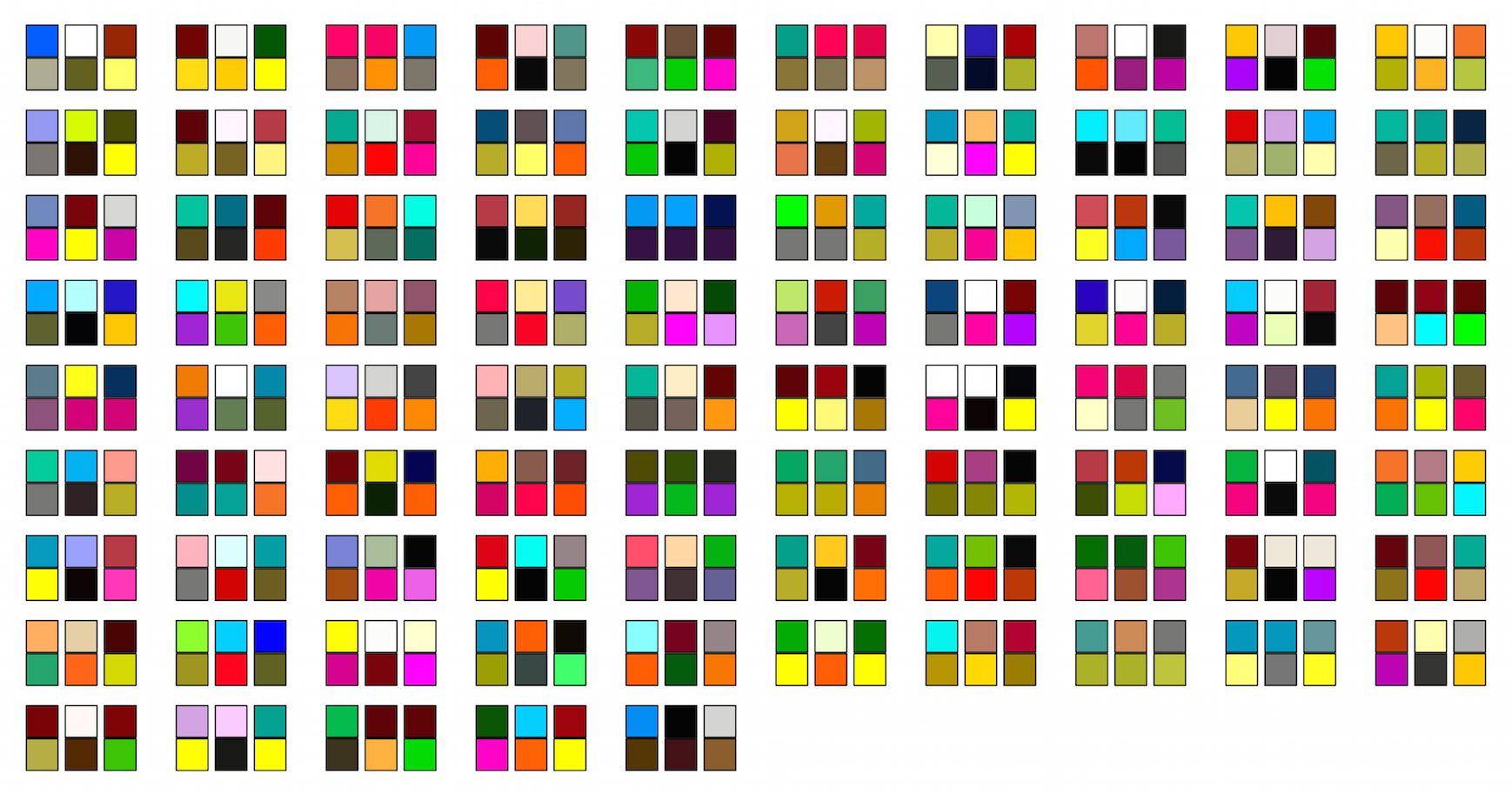

Supplement: S2 Fig — One large rectangle represents one participant. Top line of three squares in each rectangle represents most preferred colours while bottom line of three squares represents least preferred colours. Small squares represent a colour choice for each context (columns left to right: general, walls, t-shirt). (TIF) [file pone.0152194.s003.tif]
